# A Pan-HPV Vaccine Based on Bacteriophage PP7 VLPs Displaying Broadly Cross-Neutralizing Epitopes from the HPV Minor Capsid Protein, L2

**DOI:** 10.1371/journal.pone.0023310

**Published:** 2011-08-17

**Authors:** Ebenezer Tumban, Julianne Peabody, David S. Peabody, Bryce Chackerian

**Affiliations:** Department of Molecular Genetics and Microbiology, University of New Mexico School of Medicine, Albuquerque, New Mexico, United States of America; Karolinska Institutet, Sweden

## Abstract

**Background:**

Current human papillomavirus (HPV) vaccines that are based on virus-like particles (VLPs) of the major capsid protein L1 largely elicit HPV type-specific antibody responses. In contrast, immunization with the HPV minor capsid protein L2 elicits antibodies that are broadly cross-neutralizing, suggesting that a vaccine targeting L2 could provide more comprehensive protection against infection by diverse HPV types. However, L2-based immunogens typically elicit much lower neutralizing antibody titers than L1 VLPs. We previously showed that a conserved broadly neutralizing epitope near the N-terminus of L2 is highly immunogenic when displayed on the surface of VLPs derived from the bacteriophage PP7. Here, we report the development of a panel of PP7 VLP-based vaccines targeting L2 that protect mice from infection with carcinogenic and non-carcinogenic HPV types that infect the genital tract and skin.

**Methodology/Principal Findings:**

L2 peptides from eight different HPV types were displayed on the surface of PP7 bacteriophage VLPs. These recombinant L2 VLPs, both individually and in combination, elicited high-titer anti-L2 IgG serum antibodies. Immunized mice were protected from high dose infection with HPV pseudovirus (PsV) encapsidating a luciferase reporter. Mice immunized with 16L2 PP7 VLPs or 18L2 PP7 VLPs were nearly completely protected from both PsV16 and PsV18 challenge. Mice immunized with the mixture of eight L2 VLPs were strongly protected from genital challenge with PsVs representing eight diverse HPV types and cutaneous challenge with HPV5 PsV.

**Conclusion/Significance:**

VLP-display of a cross-neutralizing HPV L2 epitope is an effective approach for inducing high-titer protective neutralizing antibodies and is capable of offering protection from a spectrum of HPVs associated with cervical cancer as well as genital and cutaneous warts.

## Introduction

Human papillomaviruses (HPVs) are non-enveloped viruses with an icosahedral capsid of 55–60 nm in diameter. The HPV capsid is composed of 360 copies of the major capsid protein (L1) and up to 72 copies of the minor capsid protein (L2) which encapsidate a double-stranded circular DNA genome of about 8 kb [1]–[4]. HPVs can be divided into over 100 genotypes with distinct tissue tropisms [5]. Infection with cutaneous HPV types (such as HPV1, 2, 3, 5, 8, & 10) causes benign cutaneous warts on normal individuals and on patients suffering from epidermodysplasia verruciformis (HPV5). Mucosal HPV types include those that infect the oral cavity (HPV13), part of the respiratory tract (especially the throat; HPV6 & 11), and the epithelium of the anogenital region [6], [7]. The anogenital HPVs are further divided into low-risk and high-risk or carcinogenic HPVs. The low-risk HPVs (such as HPV6, 11, & 42) cause genital warts whereas the 15–18 high-risk HPVs are associated with cervical cancer. Of the high-risk HPVs, HPV16 and HPV18 account for approximately 70% of cases of cervical cancer [6], [7].

Currently, two prophylactic vaccines have been licensed to prevent HPV infection. Cervarix® (GlaxoSmithKline) and Gardasil® (Merck) contain virus-like particles (VLPs) derived from HPV16 and HPV18 L1; in addition to these VLPs Gardasil® also contains HPV6 and HPV11 L1 VLPs. Both vaccines are highly immunogenic and elicit high titer, long-lasting neutralizing antibody responses. One limitation of these vaccines is that L1 VLPs by and large induce type-specific antibody responses, meaning that the current L1 VLP-based vaccines provide protection against a subset of HPV types associated with cancer and only offer limited cross-protection against other clinically important carcinogenic HPVs [8]–[10]. Thus, even vaccinated women will need to be monitored for HPV infection-related lesions caused by HPV types not included in the current vaccines. A nonavalent HPV vaccine consisting of L1 VLPs derived from HPV6, 11, 16, 18, 31, 33, 45, 52, and 58 is being developed by Merck and is currently in clinical trials [11]. It is predicted that this vaccine will provide ∼90% protection from infection with high-risk types, assuming that the vaccine is effective and the increase in VLP types does not diminish the antibody responses to the four original VLP types [7]. However, it is unlikely that an L1-VLP based vaccine could provide complete (100%) protection from all carcinogenic HPVs.

As an alternative to multitype L1-VLP vaccines, we and others have been interested in developing pan-HPV vaccines targeting the HPV minor capsid protein L2. Although L2 is not required to make VLPs, it is an essential component of infectious virions, playing roles in virus entry and trafficking of the viral genome to the nucleus [2]. In its context on native virions, L2 is poorly immunogenic. Neither natural infection nor immunization with HPV L1/L2 VLPs elicits anti-L2 antibody responses. However, vaccination with recombinant L2 protein or peptides derived from L2 results in the production of neutralizing antibodies that are protective in animal models [12]–[16]. This somewhat contradictory data showing that L2 is poorly immunogenic, yet is the target of neutralizing antibodies, can be explained by recent studies that shed light on the role of L2 during viral infection. Structural studies have shown that L2 is poorly exposed on the surface of virions [2]. However, it has been proposed that after the virus binds to basement membrane heparin sulfate moieties, the capsid undergoes a conformational change that exposes the amino terminus of L2 [17], [18]. Once exposed, 12 amino acids at the N-terminus of L2 are susceptible to proprotein convertase cleavage, exposing L2 epitopes near the N-terminus of the protein [19]. Antibodies against L2 can interact with this form of the virus, and prevent the stable interaction between basement membrane-bound virus and target epithelial cells [20].

Because L2 neutralizing epitopes are exposed transiently, natural HPV infection fails to elicit anti-L2 neutralizing antibody responses. Thus, it is likely that there has been little evolutionary pressure for L2 to undergo antigenic variation. Correspondingly, amino acid sequence analysis of the amino terminus of HPV L2 reveals a great degree of homology amongst HPV and even animal papillomavirus types. Immunization with L2 not only elicits antibodies that are capable of homologous neutralization, but these antibodies can also neutralize heterologous HPV types [21], [22]. For example, Pastrana et al. demonstrated that an N-terminal peptide (amino acids 1–88) of bovine papillomavirus type 1 (BPV1) can elicit neutralizing antibodies to BPV1 as well as low titer cross-neutralizing antibodies to heterologous HPVs such as HPV6, HPV16, HPV18, and HPV31 [23]. Similar results have been obtained using short synthetic peptide vaccines. In the late 1990s Kawana et al. observed that a peptide representing HPV16 L2 amino acids 108–120 elicited cross-neutralizing antibodies against both “low-risk” and “high-risk” HPVs [24], [25]. Moreover, recent studies have shown that sera generated from immunization with HPV16 L2 peptides can cross-neutralize multitude of HPV pseudoviruses (PsVs) ranging from cutaneous- to mucosal-associated HPV types [26], [27]. Nevertheless, it is worth noting that in all these studies high neutralization titers were only observed with BPV1, HPV16, and HPV18; neutralization titers against other heterologous HPV PsVs were low. Thus, in general, the L2 capsid protein is less immunogenic and induces lower titer neutralizing antibodies compared to VLPs comprised of L1 [22], [28], [29].

Numerous studies have attempted to increase the immunogenicity of L2. For example, synthetic peptides of cross-neutralizing epitopes from HPV16 L2 have been conjugated to keyhole limpet hemocyanin (KLH) or linked to a ubiquitous T helper epitope [12], [26], [27], [30]. Other studies have displayed these epitopes genetically on the surface of recombinant BPV1 L1-VLPs [30], [31]. Both strategies increased serum antibody titers but, in general, the antibodies induced by these peptides only cross-neutralized subsets of HPV types. As an approach to broaden the cross-neutralizing antibodies elicited by L2, Jagu et al., designed a concatenated multimeric L2 fusion protein, which contains amino-terminal L2 peptides derived from 3 to 22 HPV types [28]. These vaccines elicited extremely broad neutralizing antibody responses, although adjuvant was required for optimal neutralization of HPV PsVs in vivo.

One of the reasons that the HPV L1-VLP vaccine is so effective is that it presents the target antigen (L1) in a highly multivalent format that is characteristic of the structure of virus particles. VLPs induce strong antibody responses because the regularity of their capsid structure presents viral epitopes as dense, highly repetitive arrays, which are strongly stimulatory to B cells [32]. As such, VLPs have been exploited as platforms to increase the immunogenicity of molecules that are poorly immunogenic in their native context [32]. We have recently shown that the genetic display of a broadly cross-neutralizing epitope of HPV16 L2 (epitope 17–31) on VLPs of the RNA bacteriophage PP7 elicits high titer antibodies capable of protecting mice against vaginal challenge with PsV16 and PsV45, a phylogenetically divergent oncogenic-HPV [33]. Given the immunogenicity of this 16L2 PP7 VLP and the level of protection against the two PsVs, we decided to explore the immunogenicity of other types of HPV L2 peptides (17–31) on the same PP7 display platform with the aim of identifying an immunogen with the broadest protection and cross-reactivity against other HPV types. Here, we demonstrate the utility of this approach in eliciting antibody responses that can protect *in vivo* against HPV PsVs that represent diverse HPV-types associated with three different clinical manifestations; cervical cancer, genital warts, and cutaneous warts.

## Results

### HPV L2 peptides displayed on PP7 VLPs are highly immunogenic

**Figure 1:**
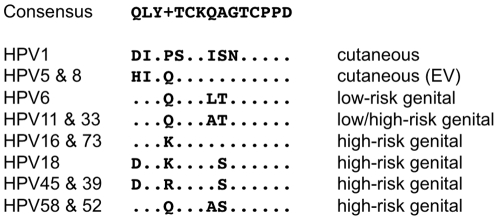
Amino acid sequence alignment of L2 (residue 17–31, or equivalent) from selected carcinogenic, genital, and/or cutaneous HPV types. The consensus sequence was derived from an alignment of 15 carcinogenic HPVs, 2 genital HPVs, 4 cutaneous HPVs plus 2 animal papillomaviruses using ClustalW2. Recombinant L2 PP7 VLPs displaying these peptide sequences were constructed. Amino acids are shown in single-letter code, dots indicate that the amino acid is identical to consensus sequence, and amino acid differences from the consensus are shown for each HPV type. A plus (+) symbol indicates that there is no consensus amino acid at this position.

**Figure 2:**
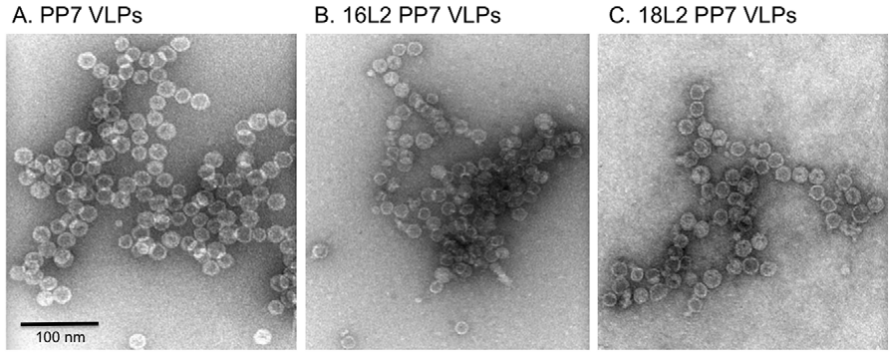
Transmission electron micrographs of selected PP7 VLPs. Shown are A) wild-type PP7 VLPs, B) 16L2 PP7 VLPs, and C) 18L2 PP7 VLPs. VLPs were visualized at a magnification of 40,000×.

**Figure 3:**
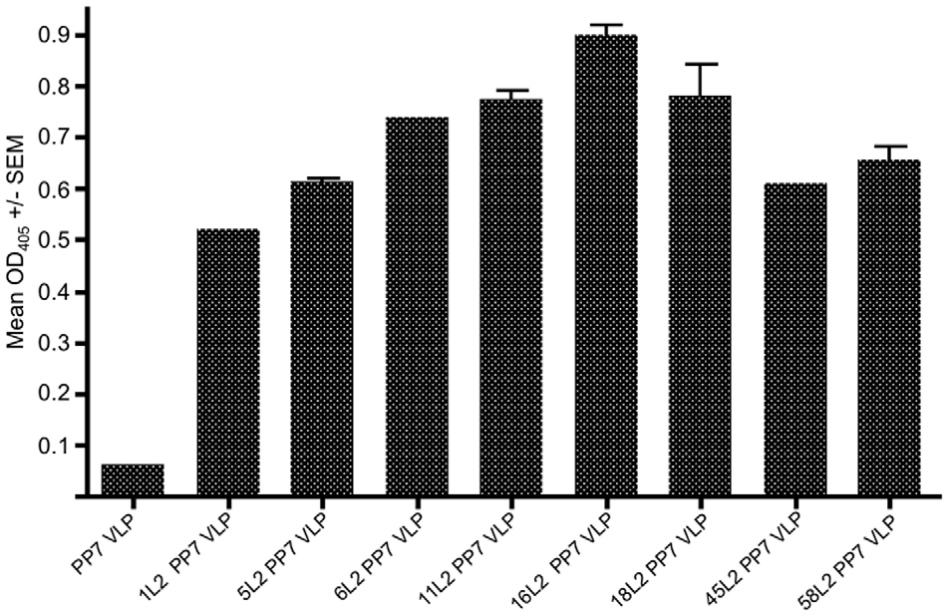
Reactivity of the RG-1 monoclonal antibody with recombinant L2 PP7 VLPs. 500 ng of wild-type PP7 VLPs or L2 PP7 VLPs were used to coat ELISA plates. Binding of a 1∶5,000 dilution of RG-1 was detected using a horseradish peroxidase-conjugated goat anti-mouse IgG secondary antibody followed by development with 2,2′-azino-bis(3-ethylbenzthiazoline-6-sulfonic acid) (ABTS). Reactivity was determined by measuring the mean optical density (OD) values at 405 nm. Error bars indicate standard error of the mean (SEM) of duplicate wells.

We have previously shown that a peptide derived from amino acids 17–31 of HPV16 L2 can be genetically inserted into a surface-exposed loop of the PP7 bacteriophage coat protein and that this recombinant coat protein self-assembles into VLPs that display the L2 peptide on their surface [33]. This epitope from L2 was selected because it is highly conserved amongst HPV types and it has been shown to contain one or more highly cross-reactive neutralizing epitopes [12], [26]. Sequences encoding this region of L2 from other HPV types were genetically inserted into PP7 coat using the same strategy, and these constructs were used to generate seven additional L2 recombinant VLPs representing sequences from carcinogenic, genital, and cutaneous HPV types (shown in Figure 1). The ability of the purified L2 PP7 coat proteins to assemble into VLPs was observed using a transmission electron microscope (TEM). As shown in Figure 2, recombinant coat protein displaying the HPV16 and HPV18 L2 peptides assembled into VLPs with similar morphology as wild-type PP7 VLPs. Additionally, the exposure of L2 peptides on the surface of VLPs was confirmed by ELISA using RG-1, an anti-L2 monoclonal antibody whose epitope maps to this region of HPV16 L2 [26]. The RG-1 mAb bound to all of the L2 PP7 VLPs, but not with wild-type PP7 VLP, demonstrating surface exposure of the epitope (Figure 3). The cross-reactivity of RG-1 which we observed was much greater than has been previously reported [26]. It is unclear whether this enhanced binding is a consequence of the multivalency of display or the possibility that the VLPs may display the L2 epitope in a favorable conformation allowing cross-reactivity.

**Figure 4:**
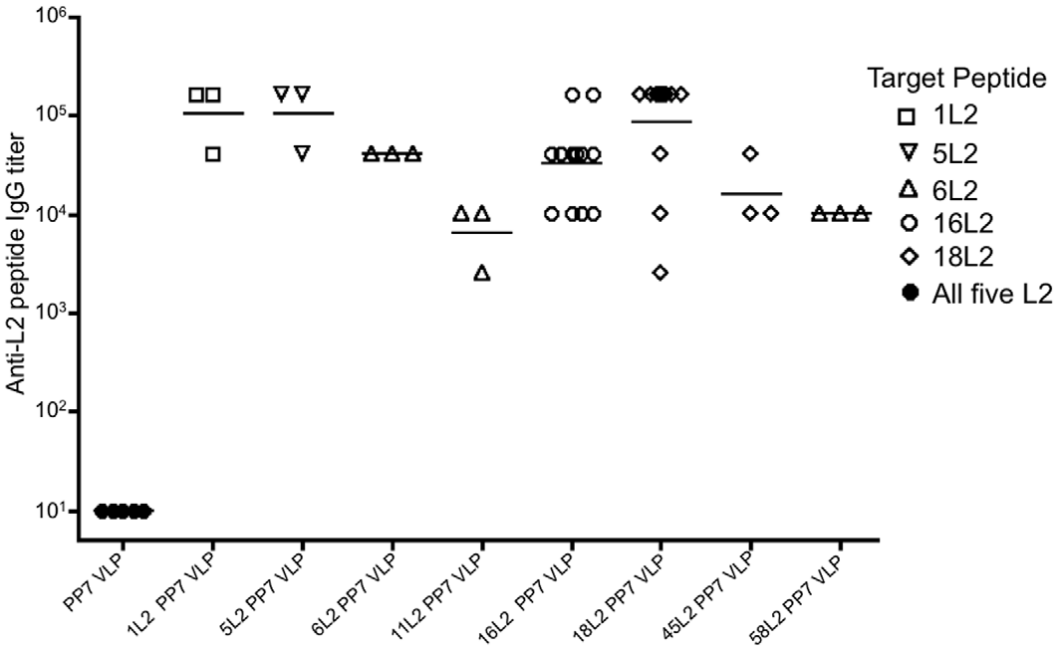
Serum IgG antibody responses in mice immunized with individual L2 PP7 VLPs. Mice were immunized i.m. twice with 5 µg of PP7 or L2 PP7 VLPs with IFA and their sera collected 2 weeks after the last immunization. Serum anti-L2 IgG titer was determined by end-point dilution ELISA against streptavidin-conjugated HPV L2 peptides (amino acid 14–40) from HPV1 (squares), HPV5 (inverted triangles), HPV6 (upright triangles), HPV16 (white-filled circles), and HPV18 (diamonds). Black-filled circles denote reactivity of PP7 sera with the different five L2 peptides. Each datum point shows antibody titer from each mouse and lines represent the geometric mean titers for each group.

To test the immunogenicity of the recombinant L2 PP7 VLPs, mice were immunized twice with 5 µg of L2 PP7 VLPs or wild-type PP7 VLPs. In this experiment, immunizations were performed using incomplete Freund's adjuvant (IFA). Sera were analyzed for anti-L2 IgG by end-point dilution ELISA using five synthetic disulfide-constrained L2-peptides representing amino acids 14–40 from HPV1, 5, 6, 16, and 18 as target antigens. Based on amino acid sequence similarities in this region of L2, the 6L2 peptide was used as target antigen for both 11L2 PP7 VLP- and 58L2 PP7 VLP-derived sera and the 18L2 peptide was used as a target antigen for the 45L2 PP7 VLP-derived sera. As shown in Figure 4, all of the L2 PP7 VLPs analyzed in this study were very highly immunogenic. Geometric mean anti-L2 IgG titers in each group ranged from 10^4^ to 10^5^ (except 11L2 PP7 VLP-derived sera which was somewhat lower; this may reflect the fact that we used a 6L2 peptide to detect anti-L2 antibody responses). No reactivity was observed with PP7 VLP-derived serum with all the synthetic L2 peptides tested. Thus, L2 peptides displayed on the surface of PP7 VLPs display the high immunogenicity that is characteristic of other VLP-displayed antigens.

### Sera generated from HPV L2 peptides is cross-reactive with L2 from other types of HPVs

**Figure 5:**
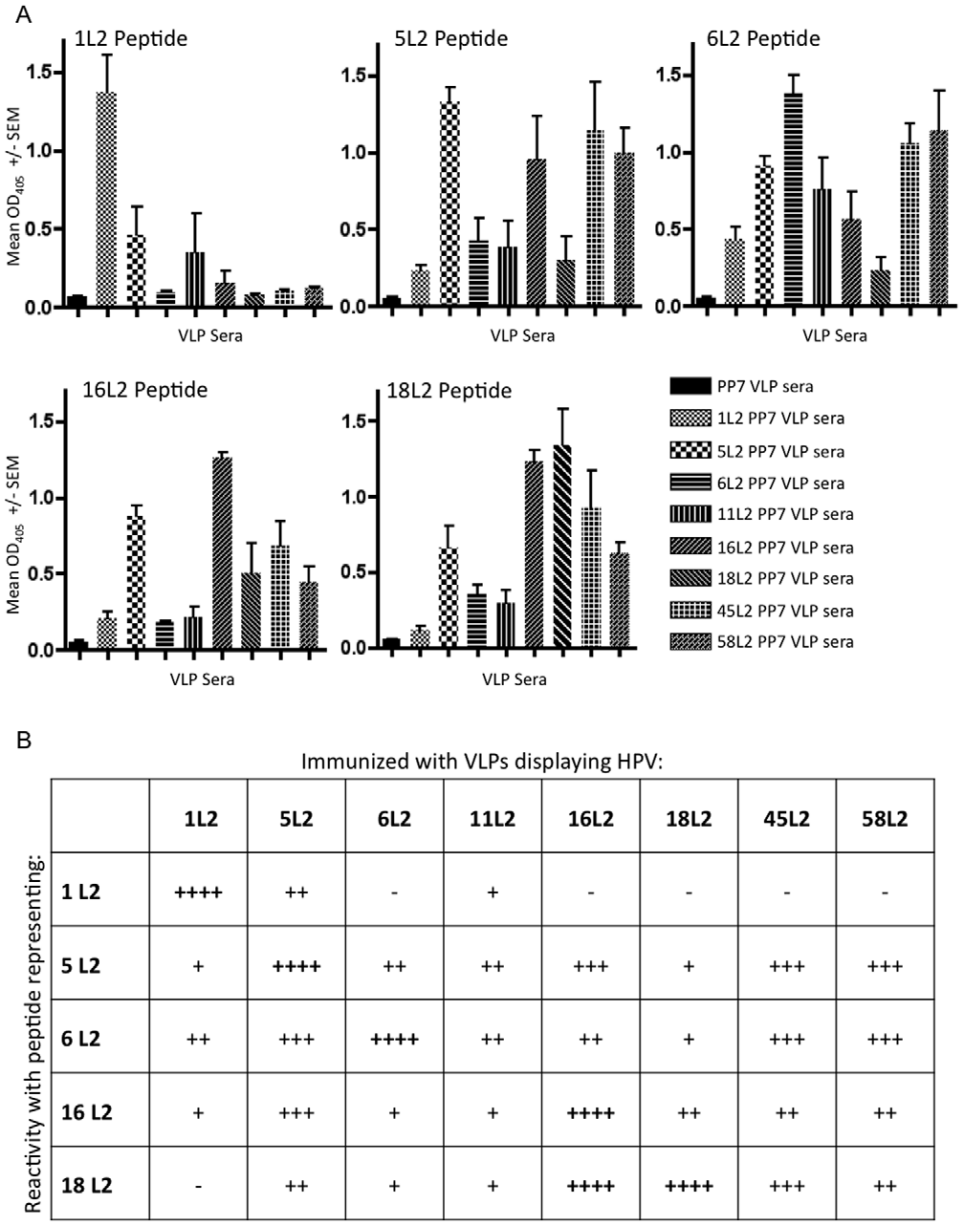
Cross-reactivity of sera elicited upon immunization with individual L2 PP7 VLPs with diverse L2 peptides. (A) ELISA plates were coated with the streptavidin-conjugated L2 peptides described in Figure 4 and reacted with a 1∶160 dilution of serum from mice immunized with wild-type PP7 VLPs or L2 PP7 VLPs followed by secondary antibody. Shown are the averages of the optical density (OD_405_) values of sera from three individual mice in each group. Error bars represent SEM. (B) A summary of these results. “++++” indicates a mean OD_405_>1.2, “+++” indicates a mean OD_405_ between 0.8 and 1.2, “++” indicates a mean OD_405_ between 0.4 and 0.8, “+” indicates a mean OD_405_ between 0.2 and 0.4, and “−” indicates a mean OD_405_ below 0.2.

As one of our goals was to generate an immunogen with the broadest cross-reactivity against other HPV types, we tested the ability of sera from mice immunized with L2 PP7 VLPs to react with all five synthetic HPV L2 peptides described above (Figure 5). At the dilution that we tested (1∶160), mice immunized with 5, 16, 45, and 58L2 PP7 VLPs showed the broadest cross-reactivity against the peptides that we tested. Sera from mice immunized with 6, 11, and 18L2 PP7 VLPs were somewhat less cross-reactive. Not surprisingly (given the sequence difference of this region), the least cross-reactivity was observed with the sera from 1L2 PP7 VLP immunized mice. Likewise, only 5L2 PP7 VLP-derived sera, and to some extent 11L2 PP7 VLP-derived sera cross-reacted with the HPV1 L2 peptide.

### Single L2 PP7 VLPs cross-protect mice from vaginal challenge with PsV

**Figure 6:**
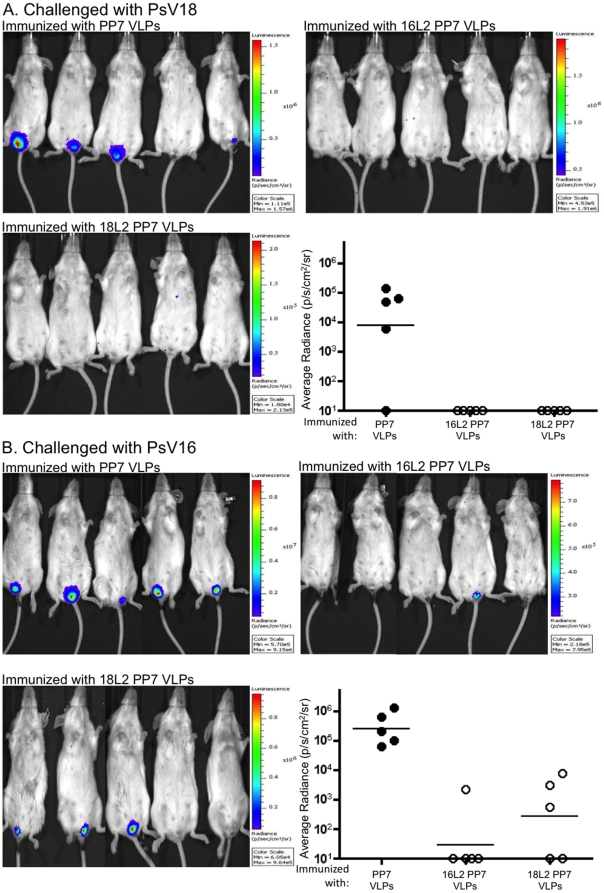
Mice immunized with 16L2 PP7 VLPs or 18L2 PP7 VLPs are protected from vaginal challenge with HPV18 and HPV16 PsV. Groups of 5 BALB/c mice were immunized i.m. twice with 5 µg of PP7 VLPs, 16L2 PP7 VLPs or 18L2 PP7 VLPs with IFA. Three weeks after the second immunization, mice were vaginally challenged with 1.3×10^5^ (PsV18) or 3.0×10^6^ (PsV16) IU of PsV. Forty-eight hours later, luciferin was instilled vaginally and images were taken 3 minutes post-luciferin instillation. Images showing the magnitude of vaginal infection with PsV18 or PsV16 are shown in panels A and B, respectively. The colors reflect the intensity of luciferase expression. Colors are scaled for each image and shown to the right of the image. Quantitative data (shown in each panel) was extracted by drawing equally sized regions of interests surrounding the site of PsV instillation and determining average radiance (p/s/cm^2^/sr) by using Living Image 3.2 software. Background radiance (determined by gating on another region of the mouse) was subtracted from this value. Black-filled circles denote mice immunized with control wild-type PP7 VLPs and white-filled circles denote mice immunized with either 16L2 PP7 VLPs or 18L2 PP7 VLPs. Lines reflect the geometric mean radiance for each group.

Since the reactivity level of 16L2 PP7 VLP sera with 16L2 peptide was similar to that with 18L2 peptide, we were interested to know if mice immunized with 16L2 PP7 VLP will be cross-protected against genital challenge with PsV18; we were also interested to know how efficient this protection would be compared to the protection provided by immunization with 18L2 PP7 VLPs. We assessed whether L2 PP7 VLPs could protect mice from HPV challenge using the HPV pseudovirus (PsV) mouse genital challenge model developed by Roberts and colleagues [34]. After two immunizations with either 16L2 or 18L2 PP7 VLPs, or control non-recombinant wild-type PP7 VLPs, mice were vaginally challenged with HPV16 or HPV18 PsVs carrying an encapsidated luciferase reporter gene. Infection was detected by measuring the expression levels (bioluminescence) of the luciferase gene encapsidated in the HPV PsV. As shown in Figure 6, vaccination with either 16L2 or 18L2 PP7 VLPs completely protected mice from PsV18 infection. Similarly, immunization with 16L2 and 18L2 PP7 VLPs substantially protected mice from genital infection with PsV16 (the geometric mean luciferase signal was 10,000- and 1000-fold lower, respectively, compared to mice immunized with PP7 VLPs). These results are consistent with the observed cross-reactivity of 16L2 PP7 VLP sera and 18L2 PP7 VLP sera with 18L2 and 16L2 peptides, respectively (Figure 5).

### Sera generated from mixed HPV L2 PP7 VLPs is highly immunogenic and induces broadly reactive anti-L2 IgG antibodies

**Figure 7:**
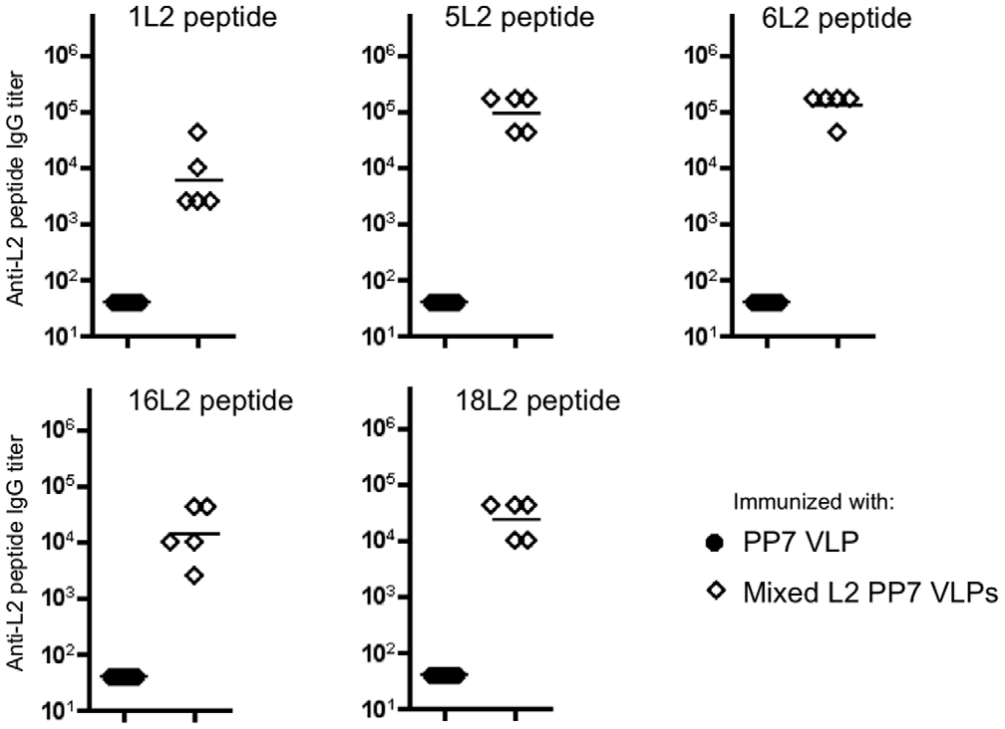
Immunization with a mixture of L2-PP7 VLPs induces broad anti-L2 IgG responses. Shown is the reactivity of sera from mice immunized with mixed L2 PP7 VLPs to L2 peptides. Mice were immunized i.m. three times with 10 µg of wild-type PP7 VLPs or 10 µg of mixed L2 PP7 VLPs. Sera were collected 2 weeks after the last immunization and serum anti-L2 IgG titer was determined by end-point dilution ELISA against streptavidin-conjugated HPV L2 peptides (amino acid 14–40) from HPV1, HPV5, HPV6, HPV16, and HPV18. Black-filled circles denote mice immunized with PP7 VLPs and diamonds denote mice immunized with mixed L2 PP7 VLPs.

In an attempt to broaden the protection conferred by vaccination, we also tested the immunogenicity of a combination vaccine consisting of all eight L2-PP7 VLPs that were constructed. Mice were immunized with a total of 10 µg of a mixture of 1L2, 5L2, 6L2, 11L2, 16L2, 18L2, 45L2, and 58L2 PP7 VLPs (mixed L2 PP7 VLPs). As shown in Figure 7, the mixed L2 PP7 VLPs elicited antibodies with broad reactivity to all five synthetic HPV L2 peptides that we tested. Geometric mean anti-L2 peptide IgG end-point dilution titers ranged from a low of 2,560 (for the 1L2 peptide) to >10^5^ (for the 6L2 peptide). The trend of reactivity was similar to that observed with single L2 PP7 VLPs. The highest reactivity was observed with both 5L2 and 6L2 HPV L2 peptides followed by 18L2 and 16L2 peptides, likely reflecting the cross-reactivity between induced antibodies.

### Mixed L2 PP7 VLPs protect mice from cervicovaginal challenge with diverse HPV pseudoviruses

**Figure 8:**
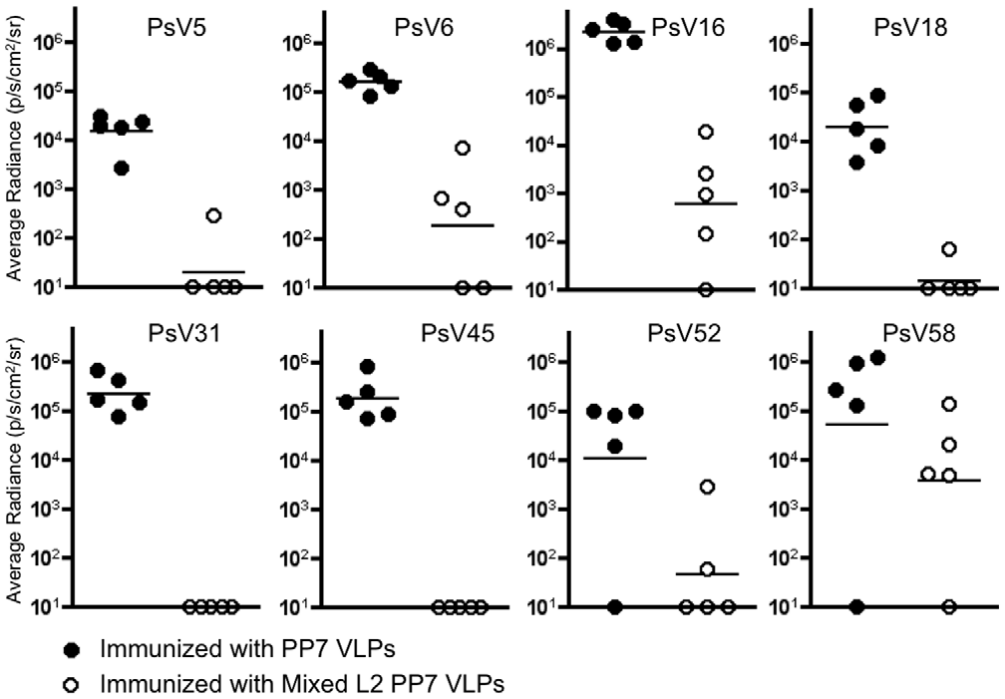
Mice immunized with mixed L2 PP7 VLPs are protected from vaginal challenge with diverse PsVs. Groups of 5 Balb/c mice were immunized i.m. three times with 10 µg of PP7 VLPs or mixed L2 PP7 VLPs. Three weeks after the last immunization, mice were vaginally challenged with 1.3×10^5^– 6.5×10^6^ IU of PsV5, PsV6, PsV16, PsV18, PsV31, PsV45, PsV52, or PsV58. Forty-eight hours later, luciferin was instilled vaginally and images were taken 3 minutes post-luciferin instillation. Average radiance (p/s/cm^2^/sr) values for each animal were determined as described in Figure 6. Black-filled circles denote mice immunized with wild-type PP7 VLPs and white-filled circles denote mice immunized with mixed L2 PP7 VLPs.

Given the fact that the broadest L2 peptide reactivity was observed upon immunization with mixed L2 PP7 VLPs, we asked whether binding of mixed anti-L2 IgG to HPV L2 peptides correlated with neutralization *in vivo*. Mice were immunized three times with mixed L2 PP7 VLPs and then were vaginally challenged with eight different HPV PsVs carrying encapsidated luciferase reporter genes, including one type (PsV31) that was not a component of our vaccine. As shown in Figure 8, mice immunized with mixed L2 PP7 VLPs were significantly protected from all eight PsV types relative to mice immunized with wild-type PP7 VLPs (all *p*<0.05, by one-tailed *t*-test). There was some variability in the degree of protection. For example, mice immunized with mixed L2 PP7 VLPs were completely protected from infection with PsV31 and PsV45. We also observed almost complete protection from infection with PsV5, PsV18, and PsV52 (except that one mouse in the control group was not infected with PsV52). Mice immunized with mixed L2 PP7 VLPs and challenged with PsV6, PsV16, and PsV58 were substantially (93–99.9% reduction of signal) protected from infection. The lack of complete protection from these PsV types may partially reflect the fact that we used somewhat higher challenge doses of these viruses. Thus, immunization with mixed L2 PP7 VLPs provides substantial and significant protection from genital challenge by diverse HPV PsVs.

### Immunization with mixed L2 PP7 VLPs protect mice from cutaneous challenge with PsV5

**Figure 9:**
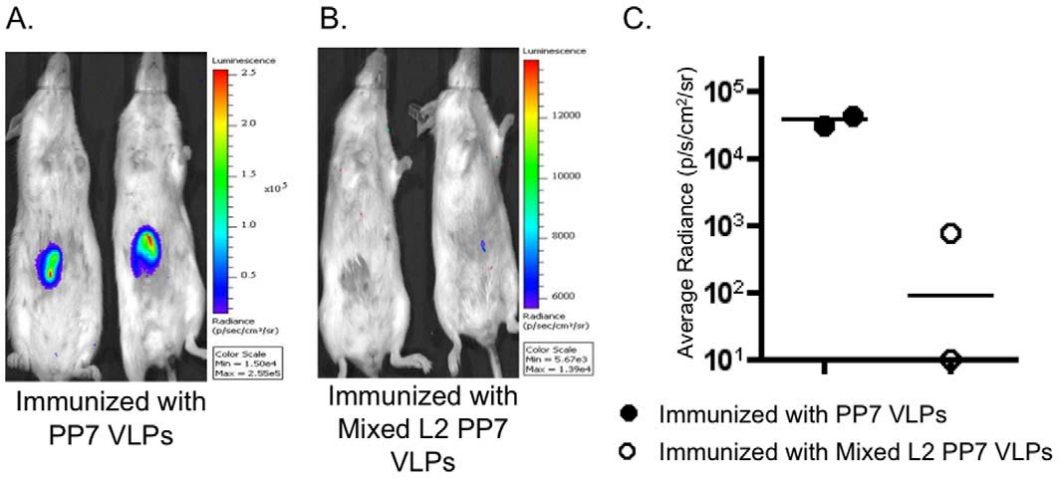
Mice immunized with mixed L2 PP7 VLPs are protected from cutaneous challenge with PsV5. Balb/c mice were immunized i.m. as described in Figure 8. Three weeks after the last immunization, mice were subcutaneously challenged in the belly with 6.0×10^5^ IU of PsV5. Three days post-challenge, mice were anesthetized and 0.7 mg luciferin was injected subcutaneously. Images of mice immunized with control PP7 VLPs and mixed L2 PP7 VLPs are shown in panels A and B, respectively. Average radiance values for the two groups are shown in panel C. Black-filled circles denote mice immunized with PP7 VLPs and white-filled circles denote mice immunized with mixed L2 PP7 VLPs.

Due to the fact that HPV5 is associated with cutaneous warts and the primary site of infection is the skin, we also tested whether our mixed L2 PP7 VLPs vaccine would also protect against cutaneous challenge with PsV5. As shown in Figure 9, we observed very efficient protection; of the two mice immunized with mixed L2 PP7 VLPs and challenged with PsV5, almost complete protection from PsV infection was observed compared to the control group that was immunized with PP7 VLP. Protection levels were similar to those observed with vaginal challenge.

## Discussion

The current licensed HPV vaccines have great potential for providing protection against the HPV types that are associated with the majority of cases of cervical cancer and genital warts. Nevertheless, the fact that these vaccines largely provide protection against only two of the 15–18 carcinogenic HPVs means that vaccinated women are still at risk for cancer and will continue to require HPV DNA and/or Pap testing. Moreover, the cost of the current vaccines may be a barrier to global implementation. Thus, it remains a priority to develop an HPV vaccine that provides broad protection against diverse HPV types and at a cost that is affordable worldwide.

The largely type-specific protection provided by L1 VLP vaccines is a significant hurdle to the development of similar L1-based vaccines that will provide protection against diverse HPVs associated with all of the clinical manifestations of infection. In contrast, immunization with L2 elicits broad (but often low titer) cross-neutralizing antibodies against multiple HPV types [12], [21]–[23], [26], [27] and therefore is a potential antigenic target for second-generation HPV vaccines. We have recently shown that the display of a broadly neutralizing epitope (L2 17–31) from HPV16 on a bacteriophage PP7 VLP elicits high titer anti-L2 IgG, which protected and cross-protected mice from vaginal challenge with PsV16 and PsV45 (an evolutionary divergent HPV), respectively [33]. Herein, we have used the same display platform to display similar epitopes from additional diverse HPV types that range from oncogenic-, genital-, to cutaneous-associated HPVs. HPV L2 peptides displayed on the PP7 VLP platform in this study were very immunogenic and elicited high titer anti-L2 antibodies after only two immunizations. An evaluation of the sera that was generated in this experiment indicated that there were different degrees of cross-reactivity with peptides derived from different HPV types. For example, the sera from mice immunized with L2 sequences from certain carcinogenic HPV types (HPV16, 45, and 58) showed high cross-reactivity with both genital- and cutaneous-associated HPV types (HPV5 and 6), and vice versa. Among the HPV L2 peptides that we used, HPV16 has the highest sequence homology at this epitope with the consensus sequence, which may explain why 16L2 PP7 VLP sera showed broad cross-reactivity. Conversely, there was little or no cross-reactivity of other L2 PP7 VLP sera with 1L2 peptide, which has only 53% sequence identity with the consensus sequence. The little cross-reactivity that was observed was mostly with 5L2 PP7 VLP sera; both HPV1 L2 and HPV5 L2 have isoleucine at position 18 of this epitope and this may suggest that isoleucine at this position is critical for reactivity with the 1L2 peptide. Based on these observations, we believe a pan-HPV vaccine from epitope 17–31 of HPV L2 must incorporate HPV1 L2 peptide given its sequence divergence from the consensus sequence. Taken together, these results provoked our use of all eight L2 PP7 VLPs as a mixed VLP vaccine. Despite the low dose (10 µg total VLPs), this mixed vaccine elicited high titer, broadly-reactive antibody responses.

Previous studies have documented low titer *in vitro* cross-neutralization with different HPV types [12], [23], [26], [28], [30], [31]. However, in most of these studies, rodents were immunized multiple times with high doses of antigen and with exogenous adjuvant. The immune responses observed in this study using PP7 VLPs as a display platform were very high given the fact that only two doses (5 µg each) were used for VLP immunizations with individual L2 PP7 VLPs. Moreover, a similar immune response was observed using a total of 10 µg of our mixed L2 PP7 VLPs. Furthermore, mixed L2 PP7 VLPs showed the broadest high-titer reactivity with diverse HPV L2 peptides. It should be noted that PP7 VLPs encapsidate an endogenous adjuvant, ssRNA, which can serve as both a TLR7 and TLR8 ligand and is likely to potentiate antibody responses [35], [36].

The immunogenicity of the mixed L2 PP7 VLPs and their broad reactivity correlated with our *in vivo* neutralization studies. Although a few studies have documented neutralization and cross-neutralization of HPV PsVs both *in vitro* and *in vivo* [12], [23], [26]–[28], [30], [31], to the best of our knowledge, this is the first study that has looked at *in vivo* protection of PsVs from 8 diverse HPV types. We observed protection from vaginal infection by all eight HPV PsVs tested as well as protection from cutaneous infection with PsV5. Protection from PsV58 infection was somewhat lower compared to other PsV types tested. This was a little surprising given the fact that efficient protection from infection was observed with PsV52, which has the same amino acid sequence as HPV58 at this epitope. Moreover, serum anti-L2 IgG titers from the two groups of mice were similar (data not shown). There are three possibilities for this discrepancy; one could be the doses of the PsV used (which were somewhat higher with PsV58; 2.3×10^6^ IU versus 1.3×10^5^ IU for PsV52). Another possibility is that this epitope on HPV58 may not be exposed as readily as that of PsV52. The third formal possibility is that mixing of the eight L2 PP7 VLPs together may lead to the induction of both neutralizing and non-neutralizing antibodies that may compete for PsV binding. However, we believe that this is unlikely given the protection levels we observed against PsV18; mixing of 18L2 PP7 VLPs with other L2 PP7 VLPs did not compromise the ability of the vaccine to protect from PsV18 challenges given the fact that mice immunized with single 18L2 PP7 VLPs or mixed L2 PP7 VLPs were equally protected from PsV18 infection.

Notably, we also observed complete protection from infection by PsV31, which was not included in our vaccine. This data provides tantalizing evidence of broad cross-neutralization, and we plan on continuing to assess the potential for broad cross-neutralization as pseudoviruses from additional HPV types become available to the scientific community. In summary, our data suggest that the mixed L2 PP7 VLPs vaccine generated in this study can serve as a pan-HPV vaccine targeting diverse HPV types. The phage-VLP approach is effective at low doses, which will be advantageous for use in resource-poor settings, where the vast majority of cases of cervical cancer arise.

## Materials and Methods

### Genetic insertion of L2 peptides into PP7 single-chain dimer

Two previously described plasmids were used for the synthesis of L2-recombinant PP7 coat protein in *E. coli* [33]. The first (p2P7K32) expresses coat protein from the lac promoter. The second plasmid type (pET2P7K32) expresses the protein from the T7 promoter and transcription terminator. Both plasmids contain an expression cassette that contains a duplicate copy of the PP7 coat protein (the so-called PP7 single-chain dimer) and produce large amounts of coat protein that assembles correctly into a VLP. PCR was used to insert HPV L2 peptides (epitope 17–31) into PP7 single-chain dimer of either plasmid as previously described [33]. Forward PCR primers encoded sequences in the following order: a KpnI restriction site, sequence encoding the L2 amino acids 17–31 (from HPV1, HPV5, HPV6, HPV11, HPV16, HPV18, HPV45, or HPV58; Figure 1), and a PP7 coat protein sequence that anneals downstream of the unique KpnI restriction site in the PP7 single-chain dimer [33]. Plasmid p2P7K32 was used as template and a primer, which anneals downstream of a BamHI restriction site of p2P7K32 (5′-GTTGTAAAACGACGGCCAGT-3′) was used as a reverse primer for all PCR amplifications. PCR fragments were cloned into either p2P7K32 or pET2P7K32 using KpnI and BamHI restriction sites. All constructs were confirmed by sequence analysis.

### Expression & purification of L2 PP7 VLPs, TEM, and antigenic characterization of L2 PP7 VLPs

L2 PP7 VLPs were made by transforming CSH41F or C41 cells (Lucigen) with plasmids p2P7K32 and pET2P7K32, respectively, containing L2 peptides from different HPV types. Constitutive expression of p2P7K32 transformed CSH41F cells was done overnight at 37°C. C41 cells transformed with pET2P7K32 were grown at 37°C until they reached an OD_600_ of 0.6. L2 PP7 protein expression was induced with 0.5 mM IPTG, grown for an additional 3 hours, and the culture was centrifuged to pellet the cells. Cell pellets were lysed and L2 PP7 VLPs were purified from the soluble fraction as previously described [33]. For TEM analysis, VLPs were adsorbed on a carbon-coated glow-discharged copper grids for 2 minutes and were negatively stained with 2% uranyl acetate for 2 minutes. VLPs were visualized using a Hitachi H7500 transmission electron microscope at a magnification of 40,000×.

To assess the antigenicity of the L2 PP7 VLPs, 500 ng of recombinant L2 PP7 VLPs or wild-type PP7 VLPs were used to coat Immulon 2 ELISA plates (Thermo Scientific) at 4°C overnight. ELISA procedure was completed as previously described [33]. Briefly, wells were blocked for 2 hours at room temperature with 0.5% non-fat dry milk in PBS buffer. Serial dilutions of monoclonal antibody (RG-1, 1∶5,000 dilution, generously provided by Richard Roden [26]) were added to each well and incubated for 2 hours at room temperature. Horseradish peroxidase (HRP)-labeled goat anti-mouse IgG (Jackson Immunoresearch, West Grove) at a dilution of 1∶5000 was applied for 1 hour as a secondary antibody. The plates were developed with 2,2′-azino-bis(3-ethylbenzthiazoline-6-sulfonic acid) (ABTS) and reactivity was determined by measuring the mean optical density (OD) values at 405 nm.

### Immunization of mice and characterization of sera for anti L2-IgG

All mice work was done in accordance with the National Institutes of Health and the University of New Mexico Institutional Animal Care and Use Committee guidelines. In mice immunized with a single recombinant L2 PP7 VLPs, groups of 3 to 13 Balb/c mice were immunized twice at a two-week interval. Immunizations were performed intramuscularly (i.m.) using 5 µg of VLPs plus incomplete Freund's adjuvant (IFA). In addition, groups of 5 Balb/c mice were immunized i.m. three times with a total of 10 µg of mixed L2 PP7 VLPs (all 8 L2 VLPs mixed together) without IFA. The mixed L2 PP7 VLPs consisted of 2 µg each of 1L2 and 5L2 VLPs, 2.5 µg of 16L2 VLPs, and 0.7 µg each of the remaining 5 VLP types. Somewhat higher amounts of 1L2, 5L2, and 16L2 VLPs were added in this mixture because 1L2 VLPs elicited antibodies that were the least cross-type reactive, and 5L2 and 16L2 VLPs elicited antibody responses that were the most cross-type reactive (Figure 5). Sera from all experimental groups were collected two weeks after the last boost and analyzed by ELISA for anti-L2 IgG.

A peptide-based ELISA was used to assess the titer of anti-L2 IgG in sera. ELISA plates were coated with 500 ng of the appropriate target peptide (representing L2 amino acids 14–40 from HPV1, 5, 6, 16, and 18; synthesized by Designer Bioscience) conjugated to streptavidin using a bifunctional cross-linker (SMPH; Thermo Scientific). The ELISA procedure was completed as described above, except that dilutions of mouse sera were used instead of mAb RG-1. Antibody titer was determined as the reciprocal of the highest sera dilution with an OD_405_ greater than 2-fold higher than control sera at the same dilution.

### HPV pseudovirus production and purification

HPV5, HPV6, HPV16, HPV18, HPV31, HPV45, HPV52, and HPV58 PsVs with encapsidated reporter plasmid (pClucf) encoding both luciferase and green fluorescence protein (GFP) genes were produced in 293TT cells as previously described [37], [38] except that matured PsVs were purified by ultracentrifugation on cesium chloride gradient at 20,000 rpm for 18 hours. PsV-infectivity titer was characterized using flow cytometry by determining the fraction of 293TT cells expressing the GFP protein. L1/L2 expression and reporter plasmids (http://home.ccr.cancer.gov/lco/packaging.htm) were generously provided by Chris Buck, Susana Pang, John Schiller, Martin Muller, and Tadahito Kanda.

### Cervicovaginal HPV PsV challenge

Prior to challenge, female Balb/c mice were given two i.m. immunizations with either 5 µg of 16L2 PP7 VLP, 18L2 PP7 VLP, or PP7 VLP in IFA, or three i.m. immunizations of 10 µg (total per immunization) of mixed L2 PP7 VLPs (all 8 L2 PP7 VLPs mixed together) or 10 µg of PP7 VLP. Three weeks after the last boost, mice were treated with 3 mg of Depo-Provera (Pharmacia Corp). Five days post-Depo-Provera treatment, mice were vaginally challenged with 1.3×10^5^– 6.5×10^6^ infectious units (IU) of the above PsVs as previously described [34], [39]. Forty-eight hours post-PsV challenge, mice were vaginally instilled with 0.4 mg of luciferin (Caliper Life Sciences) and three minutes later their images were taken (with a five minute exposure) using a Caliper IVIS Lumina II (Caliper Life Sciences). Average radiance (p/s/cm^2^/sr) was determined from the images by drawing equally sized regions of interests surrounding the site of PsV instillation. Statistical significance was determined by one-tailed paired *t*-test.

### Cutaneous HPV PsV challenge

Balb/c mice were immunized three times as described above with either PP7 VLPs or mixed L2 PP7 VLPs. Three weeks after the last immunization, they were challenged as previously described [12], [26], [40] with a few modifications. Briefly, mice were anesthetized, a patch of their belly shaved and they were challenged subcutaneously on the shaved area of the belly with 6.0×10^5^ IU PsV5 (the same dose used for cervicovaginal challenge) in 25 µl of 0.3% carboxymethylcellulose (Sigma-Aldrich). Three days post-challenged, mice were anesthetized again and 0.7 mg of luciferin was injected subcutaneously to the challenged region of the belly. Three minutes later, their images were taken at 10 minutes exposure with Caliper IVIS Lumina II (Caliper Life Sciences). Average radiance (p/s/cm^2^/sr) was determined by drawing equally sized regions of interests surrounding the site of PsV instillation.
